# Enhancing organic selenium content and antioxidant activities of soy sauce using nano-selenium during soybean soaking

**DOI:** 10.3389/fnut.2022.970206

**Published:** 2022-08-16

**Authors:** Jingru Chen, Tuo Feng, Bo Wang, Ronghai He, Yanling Xu, Peipei Gao, Zhi-Hong Zhang, Lei Zhang, Jiangyan Fu, Zhan Liu, Xianli Gao

**Affiliations:** ^1^School of Food and Biological Engineering, Jiangsu University, Zhenjiang, China; ^2^Guangdong Chubang Food Co., Yangjiang, China

**Keywords:** nano-selenium, soy sauce, antioxidant activity, *Aspergillus oryzae*, active ingredient

## Abstract

Nano-selenium has a greater potential than inorganic selenium in preventing selenium-deficiency diseases due to its higher safety. In this study, spherical nano-selenium particles (53.8 nm) were prepared using sodium selenite, ascorbic acid and chitosan. Selenium-enriched soy sauces were prepared by soaking soybean in nano-selenium and sodium selenite solutions (2–10 mg/L), respectively. Total selenium and organic selenium contents of soy sauces prepared by nano-selenium and sodium selenite were increased by 32–191-fold and 29–173-fold compared to the control (without selenium), and organic selenium accounted for over 90% of total selenium. Soy sauce prepared using 6 mg/L nano-selenium had the strongest antioxidant activities, which were 9.25–28.02% higher than the control. Nano-selenium (6 mg/L) markedly enhanced the koji's enzyme activities (9.76–33.59%), then the latter promoted the release of total phenolics (27.54%), total flavonoids (27.27%) and the formation of free amino acids (16.19%), Maillard reaction products (24.50%), finally the antioxidant activities of selenium-enriched soy sauce were enhanced.

## Highlights

- Organic selenium content of soy sauce was enhanced by nano-selenium during soybean soaking.
- Antioxidant activities of soy sauce were enhanced by nano-selenium during soybean soaking.
- Nano-selenium promoted microorganism growth and enzymes secretion during soy sauce making.
- Soybean soaking and microorganism converted nano-selenium into organic selenium.
- Enzymes from *A. oryzae* promoted formation and release of antioxidant compounds in soy sauce.

## Introduction

Selenium (Se), a micronutrient with antioxidant properties, is essential for all life forms including human beings. It often exists in membrane selenoproteins (including deiodinases, etc.) and free selenoproteins (methionine sulfur oxide reductase, glutathione peroxidase, thioxycyclin reductase, etc.) in the form of coenzyme factors or cogroups (1, 2). These selenoproteins play an important role in scavenging metabolic reactive oxygen species (ROS) in human body, which are closely related to the formation of aging, cancer, Alzheimer's disease, cardiovascular diseases, disorder in human reproduction, etc. (1, 3). Unfortunately, billions of people in the world can not intake the recommended minimum dose of selenium (60 μg/adult/day) due to the uneven distribution of selenium in land. To make matters worse, the selenium intake of approximately 1/3 people in China is less than 25 μg/adult/day (4, 5). Therefore, it is of great significance to develop commercially available and affordable selenium-enriched foods and prevent the prevailing selenium-deficiency of people in the world.

Soy sauce is a necessary fermented condiment in China, Japan, Korean and other Southeast Asia Countries. Nowadays, over 70% of soy sauce is produced using soybean, wheat flour and salt as materials according to high-salt liquid-state fermentation technology in China (1). Because *Aspergillus oryzae* 3.042 is a “generally recognized as safe” strain and secrets plentiful enzymes suitable for the production of fermented foods, which is widely used in the manufacturing of soy sauce, soybean paste, *Sufu, Douchi*, etc. (6). The main manufacturing processes of soy sauce include soybean washing, soaking, steaming, cooling, inoculating (*Aspergillus oryzae*), koji preparation, moromi preparation (22% saline:koji = 2.2:1, w/v), fermentation (6 months), etc. (7, 8). Soy sauce contains abundant small molecular peptides, free amino acids (FAAs), saccharides and Maillard reaction compounds, and it has been proved to possess a strong antioxidant activity (3, 7, 9). The cysteine, methionine and small molecular peptides are the potential carriers of selenium to form organic selenomethionine (SeMet) and selenocysteine (SeCys) *via* microbial transformation during soy sauce fermentation (10–12). Organic selenium has far higher safety and bioavailability for human compared to inorganic selenium (3, 4). Furthermore, the strong antioxidant activity of soy sauce is conducive to reduce the toxic high valence selenium to non-toxic low valence selenium or keep the stability of low valence selenium. Thus, soy sauce is an ideal carrier of selenium supplement for human. However, it is not acceptable to add inorganic selenium (Na_2_SeO_3_ and Na_2_SeO_4_) during soy sauce production due to its strong toxicity and the possible residual of inorganic selenium. Thus, it is prerequisite to enhance the selenium content in soy sauce by applying safe selenium.

Gao et al. (1) developed a selenium-enriched soy sauce by replacing soybean with selenium-enriched soybean. Results indicated that the contents of organic selenium and total selenium reached 59.6 and 79.3 μg/kg in the selenium-enriched soy sauce, respectively, which were 10.6 times and 11.1 times more than those in the ordinary soy sauce. Unfortunately, the selenium content is still difficult to meet the minimum recommended intake for human beings (50 μg/day), because the daily per capita consumption of soy sauce is approximately 10 mL in China and the other Asia countries. Furthermore, the high price of soy sauce produced by selenium-enriched soybean is not accepted by most consumers. The preparation and application of nano-selenium in foods have drawn more and more attention because of the low toxicity and high safety (13, 14). Furthermore, the antioxidant activity and immunoregulation ability of nano-selenium *in vivo* and *in vitro* are also proved by various researchers (2, 14, 15). So far, selenium-enriched soy sauce produced by nano-selenium has not been reported.

Hence, the aims of this research were to (i) develop a nano-selenium solution and selenium-enriched soy sauce using the prepared nano-selenium solution, (ii) evaluate the effect of nano-selenium on the antioxidant activities of soy sauce, (iii) clarify the enhancement mechanism of antioxidant activities of seleniums-enriched soy sauce.

## Materials and methods

### Strain and chemicals

*A. oryzae* 3.042 was supplied by Guangdong Institute of Microbiology (Guangzhou, China). Soybean, wheat flour and sodium chloride (NaCl) were purchased from a local supermarket (Zhenjiang, China). Na_2_SeO_3_, ferrozine, 2,2-diphenyl-1-picrylhydrazyl (DPPH), Folin-Cicalteu phenol reagent, 6-hydroxy-2,5,7,8-tetramethylchro-man-2-carboxylic acid (Trolox), 2,2'-azino-bis (3-ethylbenzthiazoline-6-sulphonic acid) (ABTS), gallic acid and rutin were obtained from Sigma (St. Louis, MO, USA). The other chemicals utilized in this research were ordered from Sinopharm Chemical Reagent Co., Ltd. (Shanghai, China).

### Schematic overview of the experimental program

**Figure 1:**
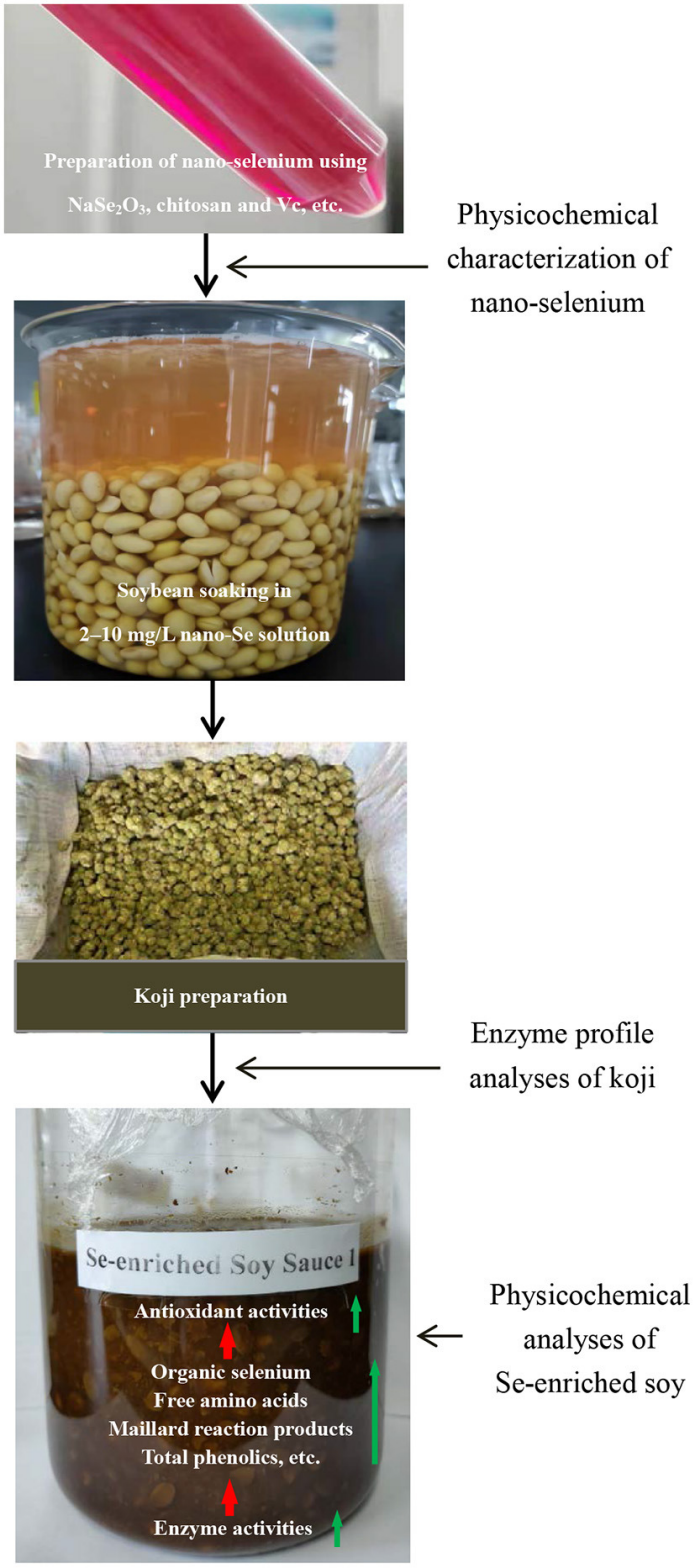
Schematic overview of the experimental program.

The experimental program of this work was shown in Figure 1, the detailed experimental methods were described in the following sections.

### Preparation of nano-selenium

Ten milliliters of 0.1 mol/L Na_2_SeO_3_ and 12 mL of 10 g/L chitosan solution (95% deacetylation degree) were placed in a 100-mL brown conical flask with a stopper. After shaking (100 r/min) in a constant temperature shaker (Changzhou Jintan Jingda Instrument Manufacturing Co., LTD, Changzhou, China) at 30 °C for 30 min, 10 mL of 0.5 mol/L ascorbic acid solution was added and distilled water was added until the total volume was 40 mL. After the mixture was evenly mixed, 0.025 mol/L (measured by selenium) red nano-selenium solution was obtained by shaking in the constant temperature shaker for 1 h at 30 °C. The resulting nano-selenium solution was kept at room temperature in the dark for further use.

### Physicochemical characterization of nano-selenium

The morphology and composition of the prepared nano-selenium were investigated by X-ray energy dispersive spectrometer (EDS) and transmission electron microscope (TEM).

### Preparation of soy sauce

Soy sauce was prepared in light of the report of Gao et al. (7) with minor modifications. Firstly, soybean was washed thrice with tap water. The soybean were soaked in tap water, ascorbic acid solution (40 μg/mL), chitosan solution (9 μg/mL), ascorbic acid solution (40 μg/mL) + chitosan solution (9 μg/mL), sodium selenite solution (2–10 mg/L, calculated by selenium) and nano-selenium solution (2–10 mg/L, calculated by selenium) at room temperature for 12 h, respectively, soy sauces produced using the above soybean were named as the control, AA, CTS, AA+CTS, SS2–10 and NS2–10. Secondly, the soaked soybeans were steamed, cooled, inoculated with *A. oryzae* 3.042 and mixed with wheat flour, separately, then the mixtures were cultured in incubators at 30 °C until the mixtures were covered by yellowish to green spores (mature koji) (8). The activities of α-amylase, glucoamylase, cellulase, acid protease, neutral protease, and pectinase of kojis were determined based on the methods reported by Gao et al. (8) and Meini et al. (16). Thirdly, the kojis were thoroughly blended with 3-fold of saline (18%, w/v) to prepare moromis in 2-L beakers, and then the beakers were sealed with plastic membrane and incubated in a 35 °C incubator. *L. plantarum* with a final concentration of 1 × 10^6^ cells/mL moromi was inoculated into all moromis on the first day. *Z. rouxii* with a final concentration of 2 × 10^6^ cells/mL moromi (pH <5.5) was introduced into all moromis on the 5th day. Finally, all soy sauces were taken on 180 d and kept at 4 °C refrigerator for analyses.

### Determination of total selenium and organic selenium contents

Atomic absorption spectrometry (AAS) and atomic fluorescence spectrometry (AFS) were utilized to determine the inorganic selenium and total selenium contents of soy sauces based on the report of Zhao et al. (17). Organic selenium content was obtained by subtracting inorganic selenium content from total selenium content.

### Determination of antioxidant activities

#### DPPH radical scavenging activity

In comparison to the positive control (ascorbic acid), the DPPH radical scavenging activity of soy sauces was determined in light of the reported method (18). The percentage of DPPH radical scavenging activity of soy sauce was calculated using the following equation:


$$\begin{array}{l} \text{DPPH radical scavenging efficiency (\%) }=\frac{A_{0}\;-\;\left(A_{1}\;-\;A_{s}\right)}{A_{0}}\\ \;\times \;100 \end{array}$$

Where A_0_ is the absorbance of blank solution containing only DPPH, A_1_ stands for the positive control with DPPH solution or soy sauce with DPPH, and A_s_ means the positive control without DPPH solution or soy sauce solution without DPPH. The results were described as ascorbic acid equivalent antioxidant capacity (μg AAE/mL soy sauce), which was designated as the quantity of ascorbic acid (in μg) with the equal antioxidant activity to 1 mL of soy sauce.

#### ABTS radical scavenging activity

Compared to the positive control (Trolox), the ABTS radical scavenging capacity of soy sauces was assessed based on the protocol reported by Lee et al. (19). The following equation was used to determine the ABTS scavenging efficiency:


$$\begin{array}{l} \text{ABTS radical scavenging efficiency }\left(\text{\%}\right)=\;\frac{A_{c}\;-\;A_{s}}{A_{c}}\;\times \;100 \end{array}$$

Where *A*_*c*_ is the absorbance of control (ABTS^∙+^ solution in the absence of the positive control or soy sauce) and *A*_*s*_ is the absorbance of sample (ABTS^∙+^ solution with the positive control or soy sauce). The resulting data were described as Trolox equivalent antioxidant capacity (TEAC, μmol TE/mL soy sauce), which was designated as the quantity of Trolox (in μmol) with the equal antioxidant activity to 1 mL of soy sauce.

### Evaluation of reducing power

The reducing power of soy sauces was determined with a spectrophotometer according to the reported method with some modifications (20). The reducing power was presented with μg ascorbic acid equivalent (μg AAE)/mL soy sauce.

### Evaluation of metal ion chelating activity

The chelating activity of soy sauces was evaluated in light of the reported approach with few modifications (21). The assay was carried out by adding EDTA solution or distilled water to replace soy sauce for the positive (Ethylenediaminetetraacetic acid, EDTA) and blank control. The following equation was used to calculate the Fe^2+^ chelating activity:


$$\begin{array}{l} \text{Metal ion chelating efficiency }\left(\text{\%}\right)=\;\frac{A_{c}\;-\;A_{s}}{A_{c}}\;\times \;100 \end{array}$$

Where *A*_*c*_is the absorbance of the control (distilled water), and *A*_*s*_ is the absorbance of sample (soy sauce or EDTA). In this assay, EDTA was utilized as the standard antioxidant, and data were exhibited as μg EDTA equivalent (μg EE)/mL soy sauce.

### Analyses of proximate indices

Total sugar and reducing sugar contents of soy sauces were determined in light of AOAC methods 992.23, 925.35 and 923.09, respectively (22). Total titratable acidity and formaldehyde nitrogen were assessed with titration methods (23). Total solids content of soy sauces was evaluated with AOAC method 990.2 (24). A volumetric titration with AgNO_3_ was used to determine the salt content (expressed as NaCl, g/L) of soy sauces based on Mohr's method (25). While non-salt soluble solids content (g/L) of soy sauces was obtained by subtracting salt content from the total solids content (105 °C until constant weight).

### Evaluation of total phenolics and total flavonoids contents

Total phenolics content of soy sauces, which was expressed as milligram gallic acid equivalents (GAE) per milliliter of soy sauce (mg GAE/mL soy sauce), was assessed using the Folin-Ciocalteu method according to the literature with minor revision (26). The total flavonoids content in soy sauce, which was expressed as milligram rutin equivalents per milligram of soy sauce (mg RE/mL soy sauce), was evaluated with a colorimetric method using rutin as the standard.

### Determination of Maillard reaction products

After a 15-fold dilution of soy sauce with distilled water, it was filtrated with micropore films (0.45 μm of pore size) before further analysis. The Maillard reaction products in soy sauce were determined using the absorbance of diluted soy sauce at 420 nm multiplying dilution multiple.

### Determination of free amino acids contents

The diluted soy sauce was filtrated with the micropore filter (0.45 μm pore size, Sangon Biotech, Shanghai, China) before FAA analysis, then the resulting filtrate was utilized to measure the FAA composition with the technique reported by Gao et al. (8) using a PICO·TAG amino acid analysis column (3.9 mm i.d. × 150 mm height; Waters Ltd., Milford, MA, USA) connected to an high-performance liquid chromatography (Waters Ltd., Milford, MA, USA). The detection was performed at the wavelength of 254 nm at 38 °C. The injection volume was 10 μL, and the flow rate of mobile phase was 1.0 mL/min. The standard amino acids (amino acid standard solution, type H, Sigma AAS18, St Louis, MO, USA) were used to determine the concentrations of amino acids in soy sauce.

### Determination of antioxidant activities of ingredients in NS6

The antioxidant activities of soluble soybean polysaccharide (37,100 μg/mL), chitosan (9 μg/mL), ascorbic acid (40 μg/mL), sodium selenite (13.14 μg/mL), nano-selenium (6 μg/mL) and free amino acids (47,590 μg/mL) in NS6 were measured to clarify the contribution of nano-selenium to the enhanced antioxidant activities of selenium-enriched soy sauce.

### Statistical analyses

All assays were carried out thrice, and all data were described as mean ± standard deviations. The obtained results were analyzed using one-way analysis of variance (ANOVA). The differences at *p* value below 0.05 were considered to be statistically significant unless otherwise specified. All calculations were performed by SPSS 15.0 (version 15.0 for Windows, SPSS Inc., Chicago, IL, USA).

## Results and discussion

### Physicochemical characterization of nano-selenium

**Figure 2:**
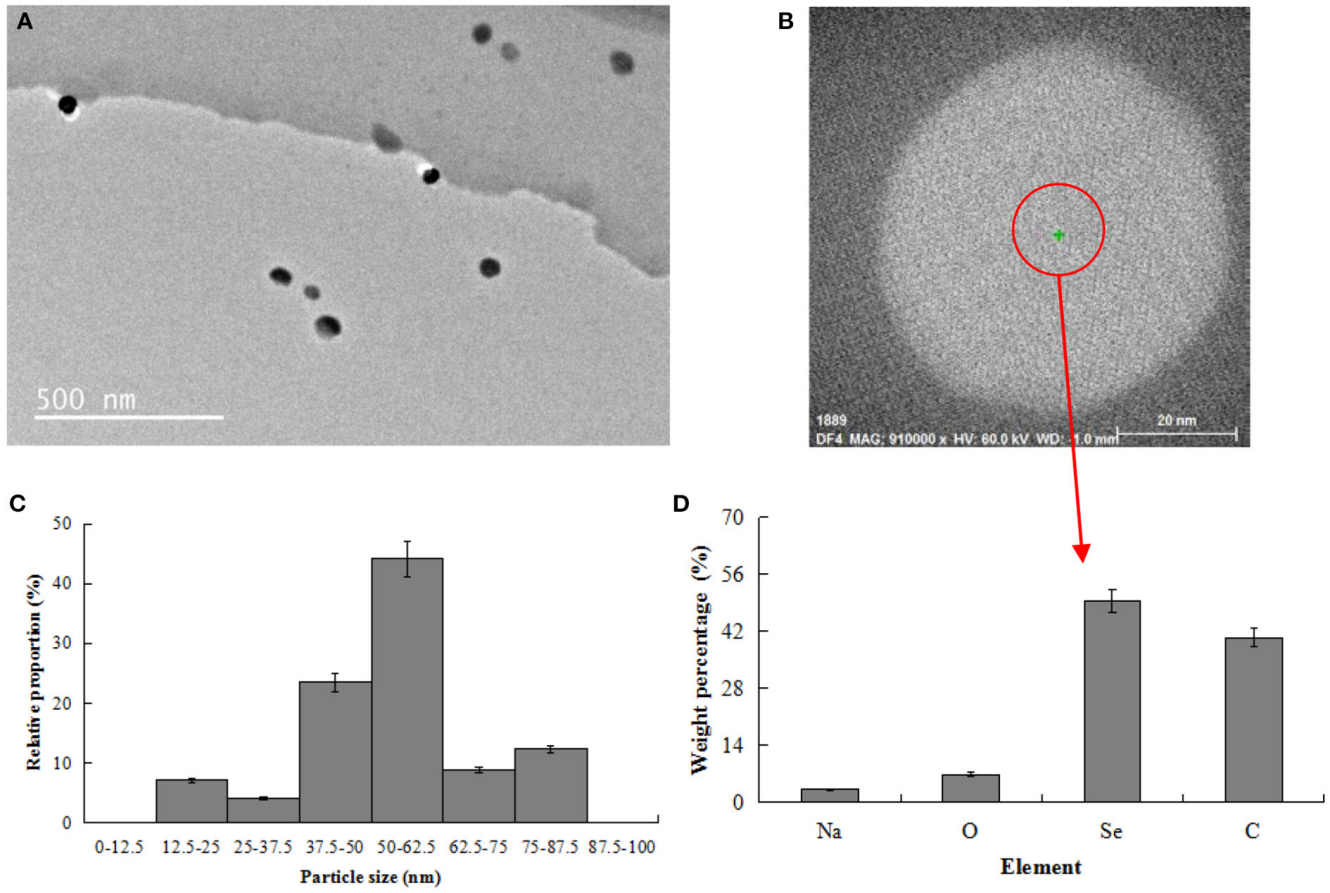
Physicochemical characterization of nano-selenium. **(A)** TEM image of nano-selenium (500 nm); **(B)** TEM image of nano-selenium (20 nm); **(C)** Particle size distribution of nano-selenium; **(D)** Element distribution of nano-selenium.

As shown in Figure 2A, the nano-selenium particles prepared in this work showed a relatively uniform dispersion state in the nano-selenium chitosan system. As shown in Figure 2B, the morphology of nano-selenium was complete and presented a regular spherical state. As shown in Figure 2C, the diameter of most spherical nano-selenium ranged from 37.5 nm to 62.5 nm, with an average diameter of about 53.8 nm. Chen et al. (27) synthesized nano-selenium particles with an average diameter of 43.2 nm and high uniformity using chitosan as template, suggesting that the nano-selenium particles prepared in the present work were successful.

As shown in Figure 2D, the nano-selenium particles contained four main elements: Se, C, O and Na, they accounted for 49.38, 40.44, 6.93, and 3.25%, respectively. C and O were derived from soft template chitosan [(C_6_H_11_NO_4_)_n_] and reduced Na_2_SeO_3_, and Na and Se were derived from reduced Na_2_SeO_3_. N element was adjacent to C and O elements, and the content of N in nano-selenium was low, which was not easy to be identified by energy analysis spectrometer. Thus, N element was not detected in this experiment. N element in nano-selenium particles prepared using modified chitosan as template was not detected as well in a previous investigation conducted by Bai et al. (15), which contained 13.12% C, 11.56% O, 49.95% Cu, and 25.37% Se. Different ingredients used in the nano-particles and detection scopes of energy analysis spectrometer were attributed to the different element compositions in these two nano-particles.

### Total selenium and organic selenium contents

The contents of total selenium (TSe), inorganic selenium (ISe) and organic selenium (OSe) in soy sauces increased with the enhancement of selenium concentrations, which were 182.74–1,079.15 μg/kg, 8.76–100.29 μg/kg and 173.98–978.86 μg/kg and significantly higher than those of the control, AA, CTS and AA+CTS (F_TSeSS2/control_ = 2,717.243, *p*_TSeSS2/control_ = 0.000, F_TSeSS4/control_ = 2,400.458, *p*_TSeSS4/control_ = 0.000, F_TSeSS6/control_ = 1,165.772, *p*_TSeSS6/control_ = 0.000, F_TSeSS10/control_ = 795.401, *p*_TSeSS10/control_ = 0.000, F_TSeNS2/control_ = 1,055.315, *p*_TSeNS2/control_ = 0.000, F_TSeNS4/control_ = 834.293, *p*_TSeNS4/control_ = 0.000, F_TSeNS6/control_ = 688.818, *p*_TSeNS6/control_ = 0.000, F_TSeNS10/control_ = 670.290, *p*_TSeNS10/control_ = 0.000, F_TSeSS2/AA_ = 2,713.554, *p*_TSeSS2/AA_ = 0.000, F_TSeSS4/AA_ = 2,399.792, *p*_TSeSS4/AA_ = 0.000, F_TSeSS6/AA_ = 1,165.621, *p*_TSeSS6/AA_ = 0.000, F_TSeSS10/AA_ = 795.356, *p*_TSeSS10/AA_ = 0.000, F_TSeNS2/AA_ = 1,054.379, *p*_TSeNS2/AA_ = 0.000, F_TSeNS4/AA_ = 834.069, *p*_TSeNS4/AA_ = 0.000, F_TSeNS6/AA_ = 688.728, *p*_TSeNS6/AA_ = 0.000, F_TSeNS10/AA_ = 670.247, *p*_TSeNS10/AA_ = 0.000, F_TSeSS2/CTS_ = 2,712.414, *p*_TSeSS2/CTS_ = 0.000, F_TSeSS4/CTS_ = 2,398.997, *p*_TSeSS4/CTS_ = 0.000, F_TSeSS6/CTS_ = 1,165.311, *p*_TSeSS6/CTS_ = 0.000, F_TSeSS10/CTS_ = 795.227, *p*_TSeSS10/CTS_ = 0.000, F_TSeNS2/CTS_ = 1,053.559, *p*_TSeNS2/CTS_ = 0.000, F_TSeNS4/CTS_ = 883.673, *p*_TSeNS4/CTS_ = 0.000, F_TSeNS6/CTS_ = 688.510, *p*_TSeNS6/CTS_ = 0.000, F_TSeNS10/CTS_ = 670.120, *p*_TSeNS10/CTS_ = 0.000, F_TSeSS2/CTS+AA_ = 2,713.269, *p*_TSeSS2/CTS+AA_ = 0.000, F_TSeSS4/CTS+AA_ = 2,398.719, *p*_TSeSS4/CTS+AA_ = 0.000, F_TSeSS6/CTS+AA_ = 1,165.106, *p*_TSeSS6/CTS+AA_ = 0.000, F_TSeSS10/CTS+AA_ = 795.132, *p*_TSeSS10/CTS+AA_ = 0.000, F_TSeNS2/CTS+AA_ = 1,053.293, *p*_TSeNS2/CTS+AA_ = 0.000, F_TSeNS4/CTS+AA_ = 883.427, *p*_TSeNS4/CTS+AA_ = 0.000, F_TSeNS6/CTS+AA_ = 688.357, *p*_TSeNS6/CTS+AA_ = 0.000, F_TSeNS10/CTS+AA_ = 670.026, *p*_TSeNS10/CTS+AA_ = 0.000; F_ISeSS2/control, AA, CTS, CTS+AA_ = 1,104.729, *p*_ISeSS2/control, AA, CTS, CTS+AA_ = 0.000, F_ISeSS4/control, AA, CTS, CTS+AA_ = 1,296.284, *p*_ISeSS4/control, AA, CTS, CTS+AA_ = 0.000, F_ISeSS6/control, AA, CTS, CTS+AA_ = 1,263.680, *p*_ISeSS6/control, AA, CTS, CTS+AA_ = 0.000, F_ISeSS10/control, AA, CTS, CTS+AA_ = 1,293.428, *p*_ISeSS10/control, AA, CTS, CTS+AA_ = 0.000, F_ISeNS2/control, AA, CTS, CTS+AA_ = 958.820, *p*_ISeNS2/control, AA, CTS, CTS+AA_ = 0.000, F_ISeNS4/control, AA, CTS, CTS+AA_ = 2,075.485, *p*_ISeNS4/control, AA, CTS, CTS+AA_ = 0.000, F_ISeNS6/control, AA, CTS, CTS+AA_ = 776.227, *p*_ISeNS6/control, AA, CTS, CTS+AA_ = 0.000, F_ISeNS10/control, AA, CTS, CTS+AA_ = 1,938.764, *p*_ISeNS10/control, AA, CTS, CTS+AA_ = 0.000; F_OSeSS2/control_ = 1,432.803, *p*_OSeSS2/control_ = 0.000, F_OSeSS4/control_ = 1,846.244, *p*_OSeSS4/control_ = 0.000, F_OSeSS6/control_ = 3,444.751, *p*_OSeSS6/control_ = 0.000, F_OSeSS10/control_ = 1,293.491, *p*_OSeSS10/control_ = 0.000, F_OSeNS2/control_ = 681.16, *p*_OSeNS2/control_ = 0.000, F_OSeNS4/control_ = 740.465, *p*_OSeNS4/control_ = 0.000, F_OSeNS6/control_ = 759.536, *p*_OSeNS6/control_ = 0.000, F_OSeNS10/control_ = 594.862, *p*_OSeNS10/control_ = 0.000, F_OSeSS2/AA_ = 1,431.408, *p*_OSeSS2/AA_ = 0.000, F_OSeSS4/AA_ = 1,845.677, *p*_OSeSS4/AA_ = 0.000, F_OSeSS6/AA_ = 3,443.892, *p*_OSeSS6/AA_ = 0.000, F_OSeSS10/AA_ = 1,293.407, *p*_OSeSS10/AA_ = 0.000, F_OSeNS2/AA_ = 680.714, *p*_OSeNS2/AA_ = 0.000, F_OSeNS4/AA_ = 740.281, *p*_OSeNS4/AA_ = 0.000, F_OSeNS6/AA_ = 759.435, *p*_OSeNS6/AA_ = 0.000, F_OSeNS10/AA_ = 594.825, *p*_OSeNS10/AA_ = 0.000, F_OSeSS2/CTS_ = 1,430.682, *p*_OSeSS2/CTS_ = 0.000, F_OSeSS4/CTS_ = 1,844.993, *p*_OSeSS4/CTS_ = 0.000, F_OSeSS6/CTS_ = 3,443.073, *p*_OSeSS6/CTS_ = 0.000, F_OSeSS10/CTS_ = 1,293.180, *p*_OSeSS10/CTS_ = 0.000, F_OSeNS2/CTS_ = 680.103, *p*_OSeNS2/CTS_ = 0.000, F_OSeNS4/CTS_ = 739.905, *p*_OSeNS4/CTS_ = 0.000, F_OSeNS6/CTS_ = 759.179, *p*_OSeNS6/CTS_ = 0.000, F_OSeNS10/CTS_ = 594.701, *p*_OSeNS10/CTS_ = 0.000, F_OSeSS2/CTS+AA_ = 1,430.501, *p*_OSeSS2/CTS+AA_ = 0.000, F_OSeSS4/CTS+AA_ = 1,844.688, *p*_OSeSS4/CTS+AA_ = 0.000, F_OSeSS6/CTS+AA_ = 3,442.806, *p*_OSeSS6/CTS+AA_ = 0.000, F_OSeSS10/CTS+AA_ = 1,293.023, *p*_OSeSS10/CTS+AA_ = 0.000, F_OSeNS2/CTS+AA_ = 679.798, *p*_OSeNS2/CTS+AA_ = 0.000, F_OSeNS4/CTS+AA_ = 739.668, *p*_OSeNS4/CTS+AA_ = 0.000, F_OSeNS6/CTS+AA_ = 759.003, *p*_OSeNS6/CTS+AA_ = 0.000, F_OSeNS10/CTS+AA_ = 594.610, *p*_OSeNS10/CTS+AA_ = 0.000) (Table 1). Meanwhile, the OSe conversion rates of all soy sauce were over 90%. Recently, Gao et al. (1) claimed that the TSe content of soy sauce prepared using selenium-enriched soybean was 79.3 μg/kg, which was far lower than that in the above selenium-enriched soy sauces, suggesting that soy sauces prepared by soybean soaking in nano-selenium solution was a more effective method to prepare selenium-enriched soy sauce. Notably, the contents of TSe, ISe and OSe in NS4, NS6 and NS10 were markedly lower than those in SS4, SS6 and SS10 (F_TSeNS4/SS4_ = 29.278, *p*_TSeNS4/SS4_ = 0.006, F_ISeNS4/SS4_ = 41.874, *p*_ISeNS4/SS4_ = 0.003, F_OSeNS4/SS4_ = 24.219, *p*_OSeNS4/SS4_ = 0.008; F_TSeNS6/SS6_ = 27.386, *p*_TSeNS6/SS6_ = 0.006, F_ISeNS6/SS6_ = 28.486, *p*_ISeNS6/SS6_ = 0.007, F_OSeNS6/SS6_ = 14.699, *p*_OSeNS6/SS6_ = 0.019; F_TSeNS10/SS10_ = 8.348, *p*_TSeNS10/SS10_ = 0.045, F_ISeNS10/SS106_ = 27.638, *p*_ISeNS10/SS10_ = 0.006, F_OSeNS10/SS10_ = 9.217, *p*_OSeNS10/SS10_ = 0.039), respectively, suggesting that sodium selenite was more conducive to being absorbed and transformed into organic selenium by soybean. Because sodium selenite had strong toxicity, selenium-enriched soy sauce prepared by nano-selenium was more acceptable due to its high safety. Furthermore, the contents of TSe, ISe and OSe in the control, AA, CTS and AA+CTS groups were low, which had no significant differences amongst the soy sauces (F_TSecontrol/AA_ = 0.007, *p*_TSecontrol/AA_ = 0.935, F_TSecontrol/CTS_ = 0.196, *p*_TSecontrol/CTS_ = 0.681, F_TSecontrol/AA+CTS_ = 0.677, *p*_TSecontrol/AA+CTS_ = 0.457, F_TSeAA/CTS_ = 0.096, *p*_TSeAA/CTS_ = 0.772, F_TSeAA/AA+CTS_ = 0.376, *p*_TSeAA/AA+CTS_ = 0.573, F_TSeCTS/AA+CTS_ = 0.080, *p*_TSeCTS/AA+CTS_ = 0.791; F_OSecontrol/AA_ = 0.007, *p*_OSecontrol/AA_ = 0.935, F_OSecontrol/CTS_ = 0.196, *p*_OSecontrol/CTS_ = 0.681, F_OSecontrol/AA+CTS_ = 0.677, *p*_OSecontrol/AA+CTS_ = 0.457, F_OSeAA/CTS_ = 0.096, *p*_OSeAA/CTS_ = 0.772, F_OSeAA/AA+CTS_ = 0.376, *p*_OSeAA/AA+CTS_ = 0.573, F_OSeCTS/AA+CTS_ = 0.080, *p*_OSeCTS/AA+CTS_ = 0.791).

**Table 1:** Selenium contents and antioxidant activities of soy sauces.

	**Total Se (μg/kg)**	**Inorganic Se (μg/kg)**	**Organic Se (μg/kg)**	**Organic selenium conversion rate (%)**	**DPPH radical scavenging activity (μg AAE/mL)**	**ABTS radical scavenging activity (μmol TE/mL)**	**Reducing power (μg AAE/mL)**	**Metal ion chelating activity (μg EE/mL)**
Control	5.61 ± 0.29^h^	0^h^	5.61 ± 0.29^h^	100^a^	769.25 ± 25.05^c^	56.48 ± 1.96^d, e^	2,693.72 ± 92.10^d^	229.24 ± 6.99^e^
AA	5.63 ± 0.37^h^	0^h^	5.63 ± 0.37^h^	100^a^	771.03 ± 23.73^c^	56.99 ± 1.71^d, e^	2,700.36 ± 83.29^d^	231.36 ± 6.39^d, e^
CTS	5.72 ± 0.34^h^	0^h^	5.72 ± 0.34^h^	100^a^	775.28 ± 24.96^c^	57.03 ± 1.41^d, e^	2,721.68 ± 111.18^d^	235.31 ± 6.27^d, e^
AA+CTS	5.79 ± 0.26^h^	0^h^	5.79 ± 0.26^h^	100^a^	779.75 ± 20.56^b, c^	57.86 ± 1.80^d, e^	2,732.45 ± 105.37^d^	237.68 ± 6.84^d, e^
SS2	216.15 ± 6.99^g^	11.13 ± 0.58^g^	205.02 ± 9.12^g^	94.85 ± 1.15^b^	784.36 ± 25.78^b, c^	57.12 ± 1.25^d, e^	2,713.53 ± 81.01^d^	240.78 ± 5.28^d, e^
SS4	427.46 ± 14.91^e^	25.36 ± 1.22^e^	402.10 ± 15.98^e^	94.07 ± 0.46^b, c^	807.61 ± 16.91^a, b, c^	59.71 ± 2.21^c, d^	2,863.17 ± 102.18^c, d^	265.34 ± 7.05^c^
SS6	632.89 ± 31.82^c^	47.41 ± 2.31^c^	585.48 ± 17.11^c^	92.51 ± 1.95^d, e^	829.74 ± 23.53^a, b^	63.84 ± 2.05^a, b^	3,049.28 ± 100.29^a, b^	279.93 ± 7.41^b^
SS10	1079.15 ± 65.93^a^	100.29 ± 4.83^a^	978.86 ± 46.87^a^	90.71 ± 1.20^f^	770.84 ± 27.70^c^	55.13 ± 1.62^e^	2,712.32 ± 107.53^d^	229.52 ± 6.44^e^
NS2	182.74 ± 9.44^g^	8.76 ± 0.49^g^	173.98 ± 11.17^g^	95.21 ± 1.20^b^	786.95 ± 19.24^b, c^	58.34 ± 2.13^c, d, e^	2,789.51 ± 77.69^c, d^	244.67 ± 6.48^d^
NS4	348.17 ± 20.54^f^	19.99 ± 0.76^f^	328.18 ± 20.53^f^	94.26 ± 0.34^b, c^	815.77 ± 31.10^a, b, c^	61.07 ± 1.54^b, c^	2,940.63 ± 96.63^b, c^	274.82 ± 8.26^b, c^
NS6	543.85 ± 35.52^d^	37.64 ± 2.34^d^	506.21 ± 31.46^d^	93.08 ± 0.30^c, d^	840.40 ± 27.31^a^	64.79 ± 1.40^a^	3,136.47 ± 115.68^a^	293.48 ± 9.55^a^
NS10	928.48 ± 61.74^b^	82.62 ± 3.25^b^	845.86 ± 59.67^b^	91.10 ± 0.37^e, f^	773.03 ± 32.47^c^	57.37 ± 1.37^d, e^	2,776.34 ± 84.91^c, d^	239.04 ± 6.80^d, e^

^a−h^* Different letters in the same column indicate the significant differences (p < 0.05); Organic selenium conversion rate = Organic Se/Total Se × 100%*.

The nano-selenium and sodium selenite were firstly adsorbed and were partially transformed into organic selenium by soybean during soaking. Part of the left nano-selenium and sodium selenite were then adsorbed and transformed into organic selenium by *A. oryze* during koji preparation and salt-tolerant bacterial during moromi fermentation. Finally, organic selenium was released into soy sauce by the degraded soybean proteins and autolyzed microorganism (10–12, 28, 29). This might be the possible reason for the high organic selenium conversion rates obtained in both selenium-enriched soy sauces.

### Influence of selenium on the antioxidant activities of soy sauces

As shown in Table 1, the DPPH radical scavenging activity (DRSA), ABTS radical scavenging activity (ARSA), reducing power (RP) and metal ion chelating activity (MICA) of soy sauces were 769.25–840.40 μg AAE/mL, 55.13–64.79 μmol TE/mL, 2,693.72–3,136.47 μg AAE/mL and 229.24–293.48 μg EE/mL, respectively, the above four antioxidant activities increased with the enhancement of selenium concentrations from 0 mg/L to 6 mg/L, then decreased with the enhanced selenium concentrations from 6 mg/L to 10 mg/L. The metal ion chelating activity and reducing power of ordinary soy sauce were previously reported as respectively 120.32 μg EE/mL and 2,239.87 μg AAE/mL (7), which were lower than the corresponding values of control determined in the present investigation. Higher ratio (4:1) of saline and koji used in the previous study led to the differences in the antioxidant activities between these two soy sauces. ANOVA indicated that ABTS radical scavenging activity, DPPH radical scavenging activity, metal ion chelating activity and reducing power of SS6 and NS6 were significantly higher than those of the control, AA, CTS and AA+CTS (F_ABTSSS6/control_ = 20.202, *p*_ABTSSS6/control_ = 0.011, F_ABTSNS6/control_ = 35.709, *p*_ABTSNS6/control_ = 0.004, F_ABTSSS6/AA_ = 19.752, *p*_ABTSSS6/AA_ = 0.011, F_ABTSNS6/AA_ = 37.370, *p*_ABTSNS6/AA_ = 0.004, F_ABTSSS6/CTS_ = 22.474, *p*_ABTSSS6/CTS_ = 0.009, F_ABTSNS6/CTS_ = 45.757, *p*_ABTSNS6/CTS_ = 0.002, F_ABTSSS6/CTS+AA_ = 14.415, *p*_ABTSSS6/CTS+AA_ = 0.019, F_ABTSNS6/CTS+AA_ = 27.707, *p*_ABTSNS6/CTS+AA_ = 0.006; F_DPPHSS6/control_ = 9.290, *p*_DPPHSS6/control_ = 0.038, F_DPPHNS6/control_ = 11.058, *p*_DPPHNS6/control_ = 0.029, F_DPPHSS6/AA_ = 9.255, *p*_DPPHSS6/AA_ = 0.038, F_DPPHNS6/AA_ = 11.029, *p*_DPPHNS6/AA_ = 0.030, F_DPPHSS6/CTS_ = 7.560, *p*_DPPHSS6/CTS_ = 0.049, F_DPPHNS6/CTS_ = 9.294, *p*_DPPHNS6/CTS_ = 0.038, F_DPPHSS6/CTS+AA_ = 7.675, *p*_DPPHSS6/CTS+AA_ = 0.049, F_DPPHNS6/CTS+AA_ = 9.444, *p*_DPPHNS6/CTS+AA_ = 0.037; F_MICSS6/control_ = 74.285, *p*_MICSS6/control_ = 0.001, F_MICNS6/control_ = 88.391, *p*_MICNS6/control_ = 0.001, F_MICSS6/AA_ = 73.920, *p*_MICSS6/AA_ = 0.001, F_MICNS6/AA_ = 87.697, *p*_MICNS6/AA_ = 0.001, F_MICSS6/CTS_ = 63.392, *p*_MICSS6/CTS_ = 0.001, F_MICNS6/CTS_ = 77.778, *p*_MICNS6/CTS_ = 0.001, F_MICSS6/CTS+AA_ = 52.660, *p*_MICSS6/CTS+AA_ = 0.002, F_MICNS6/CTS+AA_ = 67.694, *p*_MICNS6/CTS+AA_ = 0.001; F_RPSS6/control_ = 20.456, *p*_RPSS6/control_ = 0.011, F_RPNS6/control_ = 26.897, *p*_RPNS6/control_ = 0.007, F_RPSS6/AA_ = 21.490, *p*_RPSS6/AA_ = 0.010, F_RPNS6/AA_ = 28.081, *p*_RPNS6/AA_ = 0.006, F_RPSS6/CTS_ = 14.361, *p*_RPSS6/CTS_ = 0.019, F_RPNS6/CTS_ = 25.050, *p*_RPNS6/CTS_ = 0.011, F_RPSS6/CTS+AA_ = 14.231, *p*_RPSS6/CTS+AA_ = 0.020, F_RPNS6/CTS+AA_ = 20.000, *p*_RPNS6/CTS+AA_ = 0.011) and higher than those of SS2, SS4, SS10, NS2, NS4 and NS10 to some extent, but there was no significant differences in the antioxidant activities between SS6 and NS6 (F_ABTS_ = 0.439, *p*_ABTS_ = 0.544, F_DPPH_ = 0.262, *p*_DPPH_ = 0.636, F_MIC_ = 3.770, *p*_MIC_ = 0.124, F_RP_ = 0.973, *p*_RP_ = 0.380). The above results demonstrated that sodium selenite and nano-selenium with suitable concentration were capable of enhancing the soy sauce' antioxidant activities.

Wang et al. (4) claimed that the DPPH radical scavenging activity of fermented *Pleurotus eryngii* treated with selenium-enriched *Lactobacillus plantarum* at an initial level of 1 × 10^9^ CFU/mL was significantly increased by 107.33% compared with its control, suggesting that selenium played a positive role during *P. eryngii* fermentation. Ekumah et al. (5) found that the ABTS radical scavenging activity, DPPH radical scavenging activity and reducing power of mulberry wine supplemented with 100 μg selenium-enriched yeast/300 mL mulberry juice was markedly increased by 21.99, 12.06, and 3.20% compared with the control. Selenium had a low pKa (5.2), it could be used as a reducing agent in the experiment of reducing power (30), so that 99% of selenium was deprotonated (31), this further led to stronger nucleophilicity of selenium compounds and enhanced electron donor to reduce Fe^3+^. Lv et al. (32) proved that enzymatic hydrolysate of soybean had an iron chelating activity and speculated that the binding site between soybean protein hydrolysate and iron was the carboxyl group of Glu and Asp residues. Soy sauce contained abundant soybean peptides, which might contribute to the strong iron chelating activity. But the detailed reasons caused the significant differences in the antioxidant activities amongst the soy sauces needed further analyses.

### Analyses of proximate indices

As demonstrated in Table 2, the formaldehyde nitrogen (FN), reducing sugar (RS), total sugar (TS) and non-salt soluble solids (NSS) contents of soy sauces increased with the enhancement of selenium concentrations from 0 to 6 mg/L, then decreased with the enhanced selenium concentrations from 6 to 10 mg/L, which were 0.85–0.94 g/100 mL, 2.33–2.85 g/100 mL, 3.05–3.71 g/100 mL, and 156.45–181.45 g/L. The FN, RS, TS and NSS contents of ordinary soy sauce were previously reported to be 0.54 g/100 mL, 2.69 g/100 mL, 2.97 g/100 mL and 93.00 g/L, respectively (8), which were lower than the corresponding values except RS content of the control determined in the present investigation. Higher ratio (4:1) of saline and koji used in the previous study led to the differences in the above indices between these two soy sauces.

**Table 2:** Proximate indices of soy sauces.

	**Formaldehyde nitrogen (g/100 mL)**	**Total titratable acid (g/100 mL)**	**Reducing sugar (g/100 mL)**	**Total sugar (g/100 mL)**	**Maillard reaction products**	**Non-salt soluble solids (g/L)**	**Total phenolics (mg GAE/mL)**	**Total flavonoids (mg RE/mL)**
Control	0.85 ± 0.02^c^	1.09 ± 0.02^d, e^	2.37 ± 0.06^e^	3.07 ± 0.10^f^	7.55 ± 0.32^d^	158.48 ± 5.14^c^	2.07 ± 0.12^d^	0.22 ± 0.01^c^
AA	0.85 ± 0.03^c^	1.08 ± 0.03^d, e^	2.40 ± 0.05^e^	3.05 ± 0.08^f^	7.57 ± 0.24^d^	157.86 ± 4.89^c^	2.08 ± 0.15^d^	0.22 ± 0.01^c^
CTS	0.86 ± 0.02^c^	1.09 ± 0.03^d, e^	2.45 ± 0.06^d, e^	3.15 ± 0.14^e, f^	7.61 ± 0.37^d^	159.11 ± 5.43^c^	2.12 ± 0.13^c, d^	0.22 ± 0.01^c^
AA+CTS	0.86 ± 0.02^c^	1.11 ± 0.05^c, d, e^	2.45 ± 0.10^d, e^	3.16 ± 0.14^d, e, f^	7.66 ± 0.35^d^	159.37 ± 6.26^c^	2.12 ± 0.09^c, d^	0.22 ± 0.01^c^
SS2	0.87 ± 0.04^b, c^	1.15 ± 0.04^c, d^	2.59 ± 0.09^c, d^	3.30 ± 0.11^c, d, e^	7.83 ± 0.20^d^	164.23 ± 5.75^b, c^	2.19 ± 0.12^c, d^	0.23 ± 0.01^b, c^
SS4	0.89 ± 0.03^a, b, c^	1.29 ± 0.05^b^	2.70 ± 0.10^b, c^	3.48 ± 0.13^b, c^	8.17 ± 0.40^c, d^	171.79 ± 6.55^a, b^	2.35 ± 0.15^b, c^	0.24 ± 0.01^b^
SS6	0.92 ± 0.03^a, b^	1.40 ± 0.04^a^	2.83 ± 0.07^a, b^	3.64 ± 0.10^a, b^	8.63 ± 0.34^b, c^	179.72 ± 5.69^a^	2.61 ± 0.07^a^	0.27 ± 0.01^a^
SS10	0.85 ± 0.04^c^	1.05 ± 0.04^e^	2.33 ± 0.05^e^	3.08 ± 0.09^e, f^	7.51 ± 0.38^d^	156.45 ± 5.31^c^	2.09 ± 0.11^d^	0.22 ± 0.01^c^
NS2	0.88 ± 0.02^b, c^	1.18 ± 0.03^c^	2.60 ± 0.07^c^	3.37 ± 0.13^c, d^	7.98 ± 0.36^d^	163.96 ± 5.42^b, c^	2.28 ± 0.11^b, c, d^	0.23 ± 0.01^b, c^
NS4	0.90 ± 0.03^a, b, c^	1.32 ± 0.06^b^	2.75 ± 0.09^a, b^	3.50 ± 0.13^a, b, c^	8.84 ± 0.43^a, b^	171.37 ± 6.51^a, b^	2.44 ± 0.15^a, b^	0.24 ± 0.01^b^
NS6	0.94 ± 0.02^a^	1.44 ± 0.05^a^	2.85 ± 0.11^a^	3.71 ± 0.15^a^	9.40 ± 0.47^a^	181.45 ± 6.23^a^	2.64 ± 0.10^a^	0.28 ± 0.01^a^
NS10	0.87 ± 0.03^b, c^	1.08 ± 0.04^d, e^	2.37 ± 0.08^e^	3.13 ± 0.11^e, f^	7.59 ± 0.24^d^	160.55 ± 5.87^c^	2.13 ± 0.15^c, d^	0.22 ± 0.01^c^

^a−f^* Different letters in the same column indicate the significant differences (p < 0.05); the contents of ascorbic acid (40 μg/mL) and chitosan (9 μg/mL) in AA, CTS and AA+CTS are the same as those in NS6*.

ANOVA indicated that FN, RS, TS and NSS contents of SS6 and NS6 were significantly higher than those of the control, AA, CTS and AA+CTS (F_FNSS6/control_ = 16.333, *p*_FNSS6/control_ = 0.016, F_FNNS6/control_ = 60.750, *p*_FNNS6/control_ = 0.001, F_FNSS6/AA_ = 16.333, *p*_FNSS6/AA_ = 0.016, F_FNNS6/AA_ = 60.750, *p*_FNNS6/AA_ = 0.001, F_FNSS6/CTS_ = 12.000, *p*_FNSS6/CTS_ = 0.026, F_FNNS6/CTS_ = 48.000, *p*_FNNS6/CTS_ = 0.002, F_FNSS6/CTS+AA_ = 8.308, *p*_FNSS6/CTS+AA_ = 0.045, F_FNNS6/CTS+AA_ = 24.000, *p*_FNNS6/CTS+AA_ = 0.008; F_RSSS6/control_ = 74.682, *p*_RSSS6/control_ = 0.001, F_RSNS6/control_ = 44.025, *p*_RSNS6/control_ = 0.003, F_RSSS6/AA_ = 58.541, *p*_RSSS6/AA_ = 0.002, F_RSNS6/AA_ = 32.877, *p*_RSNS6/AA_ = 0.005, F_RSSS6/CTS_ = 50.965, *p*_RSSS6/CTS_ = 0.002, F_RSNS6/CTS_ = 30.573, *p*_RSNS6/CTS_ = 0.005, F_RSSS6/CTS+AA_ = 15.785, *p*_RSSS6/CTS+AA_ = 0.016, F_RSNS6/CTS+AA_ = 12.217, *p*_RSNS6/CTS+AA_ = 0.025; F_TSSS6/control_ = 48.735, *p*_TSSS6/control_ = 0.002, F_TSNS6/control_ = 37.809, *p*_TSNS6/control_ = 0.004, F_TSSS6/AA_ = 63.677, *p*_TSSS6/AA_ = 0.001, F_TSNS6/AA_ = 45.218, *p*_TSNS6/AA_ = 0.003, F_TSSS6/CTS_ = 24.334, *p*_TSSS6/CTS_ = 0.008, F_TSNS6/CTS_ = 22.347, *p*_TSNS6/CTS_ = 0.009, F_TSSS6/CTS+AA_ = 23.351, *p*_TSSS6/CTS+AA_ = 0.008, F_TSNS6/CTS+AA_ = 21.556, *p*_TSNS6/CTS+AA_ = 0.010; F_NSSSS6/control_ = 23.019, *p*_NSSSS6/control_ = 0.009, F_NSSNS6/control_ = 24.265, *p*_NSSNS6/control_ = 0.008, F_NSSSS6/AA_ = 25.469, *p*_NSSSS6/AA_ = 0.007, F_NSSNS6/AA_ = 26.616, *p*_NSSNS6/AA_ = 0.007, F_NSSSS6/CTS_ = 20.600, *p*_NSSSS6/CTS_ = 0.011, F_NSSNS6/CTS_ = 21.992, *p*_NSSNS6/CTS_ = 0.009, F_NSSSS6/CTS+AA_ = 17.360, *p*_NSSSS6/CTS+AA_ = 0.014, F_NSSNS6/CTS+AA_ = 18.751, *p*_NSSNS6/CTS+AA_ = 0.012) and higher than those of SS2, SS4, SS10, NS2, NS4 and NS10 to some extent, but there was no significant differences in the FN, RS, TS and NSS contents between SS6 and NS6 (F_FN_ = 0.923, *p*_FN_ = 0.391, F_RS_ = 0.071, *p*_RS_ = 0.804, F_TS_ = 0.452, *p*_TS_ = 0.538, F_NSS_ = 0.126, *p*_NSS_ = 0.740). The above results demonstrated that sodium selenite and nano-selenium with suitable concentration were capable of enhancing the FN, RS, TS and NSS contents of soy sauce. FN, RS, TS were the enzymatically hydrolyzed products of proteins and starch catalyzed by proteases, amylase and glucamylase during koji fermentation and moromi fermentation, and peptides, free amino acids, polysaccharides, monosaccharides, fats, etc. constitute NSS of soy sauce (7, 8). The materials, ratios of materials and manufacturing process were the same, thus the differences in FN, RS, TS and NSS contents between these two soy sauces might be caused by the differences in enzyme activities between these two soy sauces.

The change in total titratable acidity (TTA) content of soy sauces along with the increase of selenium content was similar to those of FN, RS, TS and NSS contents. The TTA contents of NS6 and SS6 were significantly higher than those of the control, AA, CTS and AA+CTS (F_SS6/control_ = 144.150, *p*_SS6/control_ = 0.000, F _NS6/control_ = 126.724, *p*_NS6/control_ = 0.000, F_SS6/AA_ = 122.880, *p*_SS6/AA_ = 0.000, F_NS6/AA_ = 114.353, *p*_NS6/AA_ = 0.000, F_SS6/CTS_ = 115.320, *p*_SS6/CTS_ = 0.000, F_NS6/CTS_ = 108.088, *p*_NS6/CTS_ = 0.000, F_SS6/CTS+AA_ = 61.537, *p*_SS6/CTS+AA_ = 0.001, F_NS6/CTS+AA_ = 65.350, *p*_NS6/CTS+AA_ = 0.001) and higher than those of SS2, SS4, SS10, NS2, NS4 and NS10 to some extent, but there was no marked difference in the TTA contents between SS6 and NS6 (F = 1.171, *p* = 0.340). Organic acids in soy sauce were the products of microbial metabolism (mainly lactic acid bacteria). An appropriate amount of selenium could promote the growth of microorganisms, while high concentration of selenium might inhibit their growth (1, 33). The change trend of microorganism number along with selenium content was consistent with the change trend of total titratable acidity content observed in Table 2.

The change in Maillard reaction products content of soy sauces along with the increased selenium content was similar to those of FN, RS, TS, NSS and TTA contents. The Maillard reaction products contents of NS6 and SS6 were significantly higher than those of the control, AA, CTS and AA+CTS (F_SS6/control_ = 16.051, *p*_SS6/control_ = 0.016, F _NS6/control_ = 31.758, *p*_NS6/control_ = 0.005, F_SS6/AA_ = 19.462, *p*_SS6/AA_ = 0.012, F_NS6/AA_ = 36.074, *p*_NS6/AA_ = 0.004, F_SS6/CTS_ = 12.361, *p*_SS6/CTS_ = 0.025, F_NS6/CTS_ = 26.865, *p*_NS6/CTS_ = 0.007, F_SS6/CTS+AA_ = 11.855, *p*_SS6/CTS+AA_ = 0.026, F_NS6/CTS+AA_ = 26.450, *p*_NS6/CTS+AA_ = 0.007). The Maillard reaction products were a kind of complex compounds formed *via* carbonyl-amine condensation between reducing sugars and carbonyl compounds and additional reactions, which were important components of soy sauce pigment, flavor and antioxidant compounds (7, 34). As mentioned above, selenium-enriched soy sauces contained more abundant free amino acids and reducing sugars, which would inevitably lead to form more Maillard reaction products and stronger antioxidant activities of selenium-enriched soy sauces.

### Total flavonoids and total phenolics contents

As shown in Table 2, the total flavonoids (TF) and total phenolics (TP) contents of soy sauces increased with the enhancement of selenium concentrations from 0 to 6 mg/L, then decreased with the enhanced selenium concentrations from 6 to 10 mg/L, which were 0.22–0.28 mg RE/mL and 2.07–2.64 mg GAE/mL. ANOVA indicated that TF and TP contents of SS6 and NS6 were significantly higher than those of the control, AA, CTS and AA+CTS (F_TFSS6/control_ = 45.326, *p*_TFSS6/control_ = 0.003, F_TFNS6/control_ = 39.947, *p*_TFNS6/control_ = 0.003, F_TFSS6/AA_ = 30.755, *p*_TFSS6/AA_ = 0.005, F_TFNS6/AA_ = 28.948, *p*_TFNS6/AA_ = 0.006, F_TFSS6/CTS_ = 33.041, *p*_TFSS6/CTS_ = 0.005, F_TFNS6/CTS_ = 30.156, *p*_TFNS6/CTS_ = 0.005, F_TFSS6/CTS+AA_ = 55.408, *p*_TFSS6/CTS+AA_ = 0.002, F_TFNS6/CTS+AA_ = 44.818, *p*_TFNS6/CTS+AA_ = 0.003; F_TPSS6/control_ = 37.500, *p*_TPSS6/control_ = 0.004, F_TPNS6/control_ = 54.000, *p*_TPNS6/control_ = 0.002, F_TPSS6/AA_ = 37.500, *p*_TPSS6/AA_ = 0.004, F_TPNS6/AA_ = 54.000, *p*_TPNS6/AA_ =0.002, F_TPSS6/CTS_ = 37.500, *p*_TPSS6/CTS_ = 0.004, F_TPNS6/CTS_ = 54.000, *p*_TPNS6/CTS_ = 0.002, F_TPSS6/CTS+AA_ = 37.500, *p*_TPSS6/CTS+AA_ = 0.004, F_TPNS6/CTS+AA_ = 54.000, *p*_TPNS6/CTS+AA_ = 0.002) and higher than those of SS2, SS4, SS10, NS2, NS4 and NS10 to some extent, but there was no notable differences found in the TF and TP contents between SS6 and NS6 (F_TFSS6/NS6_ = 0.181, *p*_TFSS6/NS6_ = 0.692; F_TPSS6/NS6_ = 1.500, *p*_TPSS6/NS6_ = 0.288).

TP in soy sauces prepared in this work was greater than that in the citrus peel koji soy sauce (35) and the soy sauce treated with ultrasound (7), and TF in soy sauces prepared in this work was higher than that of soy sauce reported by Gao et al. (7). Different raw materials, manufacturing process and NaCl concentration might cause the above marked differences in TP and TF contents. However, the content differences in TP and TF amongst soy sauces prepared in this work needed to be explored further.

### Free amino acids

As shown in Table 3, whether sodium selenite or nano-selenium treated soy sauces, the free amino acids (FAAs) contents of soy sauces increased with the enhancement of selenium concentrations from 0 to 6 mg/L, then decreased with the enhanced selenium concentrations from 6 to 10 mg/L, which ranged from 40.09 to 47.59 mg/mL. ANOVA indicated that FAAs contents of SS6 and NS6 were significantly higher than those of the control, AA, CTS and AA+CTS (F_SS6/control_ = 20.683, *p*_SS6/control_ = 0.010, F _NS6/control_ = 25.917, *p*_NS6/control_ = 0.007, F_SS6/AA_ = 20.042, *p*_SS6/AA_ = 0.011, F_NS6/AA_ = 25.141, *p*_NS6/AA_ = 0.007, F_SS6/CTS_ = 16.563, *p*_SS6/CTS_ = 0.015, F_NS6/CTS_ = 21.255, *p*_NS6/CTS_ = 0.010, F_SS6/CTS+AA_ = 15.503, *p*_SS6/CTS+AA_ = 0.017, F_NS6/CTS+AA_ = 20.097, *p*_NS6/CTS+AA_ = 0.011) and higher than those of SS2, SS4, SS10, NS2, NS4 and NS10 to some extent, but there was no significant difference in the FAAs contents between SS6 and NS6 (F = 0.299, *p* = 0.613). Specifically, the most plentiful amino acid was glutamic acid, followed by aspartic acid in all soy sauces, which were consistent with previous investigations (7, 8).

**Table 3:** Free amino acid compositions of soy sauces.

**FAAs (mg/mL)**	**Control**	**AA**	**CTS**	**AA+CTS**	**SS2**	**SS4**	**SS6**	**SS10**	**NS2**	**NS4**	**NS6**	**NS10**
Aspartic acid	4.08 ± 0.10^c, d^	4.04 ± 0.09^d^	4.09 ± 0.10^c, d^	4.09 ± 0.10^c, d^	4.17 ± 0.12^b, c, d^	4.32 ± 0.13^a, b, c^	4.44 ± 0.15^a^	4.06 ± 0.11^d^	4.18 ± 0.10^b, c, d^	4.37 ± 0.18^a, b^	4.52 ± 0.19^a^	4.11 ± 0.16^c, d^
Glutamic acid	6.96 ± 0.20^a^	6.95 ± 0.23^a^	6.96 ± 0.22^a^	6.96 ± 0.22^a^	7.10 ± 0.28^a^	7.26 ± 0.26^a^	7.38 ± 0.29^a^	6.96 ± 0.18^a^	7.15 ± 0.20^a^	7.28 ± 0.30^a^	7.40 ± 0.29^a^	6.99 ± 0.26^a^
Serine	2.48 ± 0.08^f^	2.49 ± 0.06^f^	2.48 ± 0.11^f^	2.51 ± 0.11^f^	2.64 ± 0.08^e, f^	2.88 ± 0.12^c, d^	3.13 ± 0.14^a, b^	2.43 ± 0.12^f^	2.75 ± 0.11^d, e^	2.98 ± 0.15^b, c^	3.22 ± 0.15^a^	2.52 ± 0.13^f^
Histidine	1.56 ± 0.05^c^	1.54 ± 0.04^c^	1.55 ± 0.04^c^	1.55 ± 0.04^c^	1.64 ± 0.04^b, c^	1.78 ± 0.07^a^	1.84 ± 0.05^a^	1.55 ± 0.03^c^	1.68 ± 0.07^b^	1.80 ± 0.08^a^	1.86 ± 0.06^a^	1.56 ± 0.04^c^
Glycine	1.04 ± 0.03^d, e^	1.03 ± 0.03^e^	1.05 ± 0.04^d, e^	1.05 ± 0.04^d, e^	1.09 ± 0.03^d, e^	1.20 ± 0.04^c^	1.33 ± 0.05^a, b^	1.02 ± 0.04^e^	1.11 ± 0.03^d^	1.27 ± 0.04^b^	1.36 ± 0.05^a^	1.08 ± 0.03^d, e^
Threonine	1.64 ± 0.06^e, f^	1.60 ± 0.04^f^	1.69 ± 0.05^d, e, f^	1.69 ± 0.05^d, e, f^	1.69 ± 0.05^d, e, f^	1.78 ± 0.06^b, c, d^	1.88 ± 0.07^a, b^	1.60 ± 0.06^f^	1.74 ± 0.05^c, d, e^	1.81 ± 0.07^b, c^	1.96 ± 0.09^a^	1.64 ± 0.06^e, f^
Arginine	3.94 ± 0.13^d, e^	3.94 ± 0.13^d, e^	4.03 ± 0.17^c, d, e^	4.08 ± 0.17^b, c, d, e^	4.14 ± 0.15^b, c, d^	4.28 ± 0.13^a, b, c^	4.48 ± 0.12^a^	3.83 ± 0.14^e^	4.17 ± 0.14^b, c, d^	4.34 ± 0.20^a, b^	4.53 ± 0.19^a^	3.98 ± 0.15^d, e^
Alanine	1.88 ± 0.07^f, g^	1.89 ± 0.05^f, g^	1.96 ± 0.08^e, f, g^	1.99 ± 0.08^d, e, f^	1.98 ± 0.08^d, e, f^	2.12 ± 0.07^c, d^	2.30 ± 0.10^a, b^	1.81 ± 0.09^g^	2.05 ± 0.08^c, d, e^	2.18 ± 0.06^b, c^	2.39 ± 0.10^a^	1.87 ± 0.11^f, g^
Tyrosine	1.16 ± 0.03^e^	1.17 ± 0.03^e^	1.18 ± 0.05^e^	1.18 ± 0.05^e^	1.23 ± 0.03^d, e^	1.30 ± 0.04^c, d^	1.40 ± 0.04^a, b^	1.17 ± 0.05^e^	1.26 ± 0.03^d^	1.36 ± 0.04^b, c^	1.44 ± 0.05^a^	1.17 ± 0.03^e^
Cysteine	0.08 ± 0.01^b^	0.07 ± 0.01^b, c^	0.10 ± 0.01^a^	0.10 ± 0.01^a^	0.07 ± 0.01^b, c^	0.06 ± 0.01^c, d^	0.04 ± 0.01^e, f^	0.03 ± 0.01^f, g^	0.06 ± 0.01^c, d^	0.05 ± 0.01^d, e^	0.03 ± 0.01^f, g^	0.02 ± 0.01^g^
Valine	2.54 ± 0.08^d^	2.53 ± 0.11^d^	2.55 ± 0.09^d^	2.55 ± 0.09^d^	2.64 ± 0.09^c, d^	2.70 ± 0.10^b, c, d^	2.84 ± 0.10^a, b^	2.53 ± 0.07^d^	2.68 ± 0.11^b, c, d^	2.79 ± 0.07^a, b, c^	2.90 ± 0.13^a^	2.58 ± 0.06^d^
Methionine	0.72 ± 0.02^b, c^	0.74 ± 0.03^b^	0.79 ± 0.02^a^	0.79 ± 0.02^a^	0.70 ± 0.01^c^	0.65 ± 0.02^d^	0.58 ± 0.01^e^	0.46 ± 0.01^f^	0.62 ± 0.03^d^	0.56 ± 0.02^e^	0.48 ± 0.01^f^	0.40 ± 0.01^g^
Tryptophan	0.46 ± 0.01^e^	0.45 ± 0.01^e^	0.48 ± 0.02^e^	0.48 ± 0.02^e^	0.54 ± 0.01^d^	0.60 ± 0.01^c^	0.68 ± 0.02^a^	0.45 ± 0.02^e^	0.59 ± 0.01^c^	0.65 ± 0.02^b^	0.70 ± 0.03^a^	0.46 ± 0.02^e^
Phenylalanine	2.68 ± 0.08^d, e^	2.70 ± 0.06^d, e^	2.65 ± 0.06^d, e^	2.65 ± 0.06^d, e^	2.79 ± 0.08^c, d, e^	2.97 ± 0.07^b, c^	3.14 ± 0.13^a, b^	2.61 ± 0.15^e^	2.83 ± 0.09^c, d^	3.09 ± 0.10^a, b^	3.24 ± 0.15^a^	2.65 ± 0.13^d, e^
Isoleucine	1.92 ± 0.07^d^	1.93 ± 0.09^c, d^	1.93 ± 0.07^c, d^	1.93 ± 0.07^c, d^	1.98 ± 0.04^c, d^	2.06 ± 0.09^b, c^	2.18 ± 0.08^a, b^	1.90 ± 0.05^d^	2.03 ± 0.06^c, d^	2.17 ± 0.08^a, b^	2.24 ± 0.09^a^	1.95 ± 0.06^c, d^
Leucine	3.18 ± 0.12^e, f^	3.21 ± 0.09^d, e, f^	3.23 ± 0.14^d, e, f^	3.23 ± 0.14^d, e, f^	3.38 ± 0.12^c, d, e^	3.52 ± 0.13^b, c^	3.81 ± 0.15^a^	3.13 ± 0.10^f^	3.43 ± 0.11^c, d^	3.66 ± 0.15^a, b^	3.85 ± 0.13^a^	3.28 ± 0.14^d, e, f^
Lysine	3.08 ± 0.09^c, d^	3.09 ± 0.09^c, d^	3.11 ± 0.08^c, d^	3.12 ± 0.08^c, d^	3.19 ± 0.10^b, c, d^	3.26 ± 0.12^b, c^	3.36 ± 0.08^a^	3.05 ± 0.09^d^	3.21 ± 0.07^b, c, d^	3.33 ± 0.16^a, b^	3.46 ± 0.11^a^	3.09 ± 0.10^c, d^
Proline	1.56 ± 0.06^f, g, h^	1.58 ± 0.04^f, g, h^	1.58 ± 0.08^f, g, h^	1.66 ± 0.08^e, f, g^	1.68 ± 0.05^d, e, f^	1.79 ± 0.05^c, d^	1.94 ± 0.06^a, b^	1.50 ± 0.06^h^	1.74 ± 0.05^d, e^	1.87 ± 0.09^b, c^	2.01 ± 0.09^a^	1.55 ± 0.07^g, h^
Total	40.96 ± 1.20^d, f^	40.95 ± 1.27^d, f^	41.41 ± 1.32^d, f^	41.61 ± 1.30^d, f^	42.65 ± 1.49^c, d, f^	44.53 ± 1.52^a, b, c^	46.75 ± 1.85^a^	40.09 ± 1.56^f^	43.28 ± 1.40^b, c, d^	45.56 ± 2.01^a, b, c^	47.59 ± 1.91^a^	40.90 ± 1.56^d, f^

^a−h^* Different letters in the same row indicate the significant differences (p < 0.05)*.

It was worth mentioning that the contents of cysteine and methionine in soy sauces treated by Na_2_SeO_3_ and nano-selenium were lower than those in the control, AA, CTS and AA+CTS. In microbial metabolism, the adsorbed selenium is usually converted into selenocysteine, Se-methyselenocysteine, selenomethionine, etc., and the synthesis of organic selenium usually requires the consumption of cysteine and methionine (10–12). By the same token, the contents of cysteine and methionine in the selenium treated soy sauces decreased.

### Antioxidant activities of ingredients in NS6

Because NS6 had the strongest antioxidant activities, antioxidant activities of the corresponding soluble soybean polysaccharide, chitosan, ascorbic acid, sodium selenite, nano-selenium and free amino acids were measured to calculate their contributions to the total antioxidant activities of NS6. As shown in Table 4, free amino acids had strong ABTS radical scavenging activity, DPPH radical scavenging activity, reducing power and metal ion chelating activity. Secondly, ascorbic acid and nano-selenium had strong reducing power and DPPH radical scavenging activity, and most reducing power and DPPH radical scavenging activity of nano-selenium should attributed to ascorbic acid in nano-selenium. However, due to the long fermentation time of soy sauce, ascorbic acid was easy to be oxidized and inactivated, losing the corresponding antioxidant capacity. Therefore, ascorbic acid and nano-selenium were not the main contributors for the antioxidant activities enhancement of NS6. From the four antioxidant activities of soybean polysaccharide, chitosan, sodium selenite, it could be seen that their contribution to the total antioxidant activities of NS6 were negligible. Therefore, FAAs might play an important role in enhancing the antioxidant activities of NS6.

**Table 4:** Antioxidant activities of ingredients in NS6.

	**Content (μg/mL)**	**DPPH radical scavenging activity (μg AAE/mL)**	**ABTS radical scavenging activity (μmol TE/mL)**	**Reducing power (μg AAE/mL)**	**Metal ion chelating activity (μg EE/mL)**
NS6	-	840.40 ± 27.31^a^	64.79 ± 1.40^a^	3,136.47 ± 115.68^a^	293.48 ± 9.55^a^
Soybean polysaccharide	33,500	4.81 ± 0.18^d^	0.02 ± 0.01^d^	10.58 ± 0.36^d^	nd
Chitosan	9	nd	nd	0.26 ± 0.01^f^	nd
Ascorbic acid	40	41.42 ± 1.41^c^	0.19 ± 0.01^c^	41.61 ± 1.57^c^	nd
Sodium selenite	13.14	nd	nd	0.89 ± 0.03^e^	nd
Nano-selenium	6	42.64 ± 2.15^c^	0.20 ± 0.01^c^	49.77 ± 2.34^b^	nd
FAAs mixture of NS6	47,590	67.14 ± 2.88^b^	23.55 ± 0.84^b^	54.52 ± 2.50^b^	10.26 ± 0.39^b^

^a−d^* Different letters in the same column indicate the significant differences (p < 0.05), nd: not detected*.

### Effects of selenium on enzyme activities of kojis

As shown in Table 5, whether sodium selenite or nano-selenium treated soy sauces, the activities of neutral protease, acid protease, α-amylase, cellulase, glucoamylase and pectinase in kojis increased with the enhancement of selenium concentrations from 0 to 6 mg/L, then decreased with the enhancement of selenium concentrations from 6 to 10 mg/L, which were 1,156.52–1,269.38 U/g, 185.34–244.60 U/g, 165.14–220.61 U/g, 240.17–314.57 U/g, 1,812.57–2,043.15 U/g, 204.43–256.36 U/g. ANOVA indicated that the neutral protease, acid protease, α-amylase, cellulase, glucoamylase and pectinase activities of SS6 and NS6 were significantly higher than those of the control, AA, CTS and AA+CTS (F_neutralproteaseSS6/control_ = 8.801 *p*_neutralproteaseSS6/control_ = 0.041, F_neutralproteaseNS6/control_ = 12.891, *p*_neutralproteaseNS6/control_ = 0.023, F_neutralproteaseSS6/AA_ = 7.823, *p*_neutralproteaseSS6/AA_ = 0.049, F_neutralproteaseNS6/AA_ = 11.818, *p*_neutralproteaseNS6/AA_ = 0.026, F_neutralproteaseSS6/CTS_ = 7.735, *p*_neutralproteaseSS6/CTS_ = 0.049, F_neutralproteaseNS6/CTS_ = 11.765, *p*_neutralproteaseNS6/CTS_ = 0.027, F_neutralproteaseSS6/CTS+AA_ = 7.685, *p*_neutralproteaseSS6/CTS+AA_ = 0.048, F_neutralproteaseNS6/CTS+AA_ = 11.803, *p*_neutralproteaseNS6/CTS+AA_ = 0.026; F_acidproteaseSS6/control_ = 59.442, *p*_acidproteaseSS6/control_ = 0.002, F_acidproteaseNS6/control_ = 121.931, *p*_acidproteaseNS6/control_ = 0.000, F_acidproteaseSS6/AA_ = 49.505, *p*_acidproteaseSS6/AA_ = 0.002, F_acidproteaseNS6/AA_ = 100.468, *p*_acidproteaseNS6/AA_ = 0.001, F_acidproteaseSS6/CTS_ = 46.324, *p*_acidproteaseSS6/CTS_ = 0.002, F_acidproteaseNS6/CTS_ = 97.105, *p*_acidproteaseNS6/CTS_ = 0.001, F_acidproteaseSS6/CTS+AA_ = 45.372, *p*_acidproteaseSS6/CTS+AA_ = 0.002, F_acidproteaseNS6/CTS+AA_ = 99.649, *p*_acidproteaseNS6/CTS+AA_ = 0.001; F_α−*amylaseSS*6/*control*_ = 89.072, *p*_α−*amylaseSS*6/*control*_ = 0.001, F_α−*amylaseNS*6/*control*_ = 100.051, *p*_α−*amylaseNS*6/*control*_ = 0.001, F_α−*amylaseSS*6/*AA*_ = 76.470, *p*_α−*amylaseSS*6/*AA*_ = 0.001, F_α−*amylaseNS*6/*AA*_ = 87.134, *p*_α−*amylaseNS*6/*AA*_ = 0.001, F_α−*amylaseSS*6/*CTS*_ = 71.513, *p*_α−*amylaseSS*6/*CTS*_ = 0.001, F_α−*amylaseNS*6/*CTS*_ = 81.836, *p*_α−*amylaseNS*6/*CTS*_ = 0.001, F_α−*amylaseSS*6/*CTS*+*AA*_ = 68.208, *p*_α−*amylaseSS*6/*CTS*+*AA*_ = 0.001, F_α−*amylaseNS*6/*CTS*+*AA*_ = 78.214, *p*_α−*amylaseNS*6/*CTS*+*AA*_ = 0.001; F_cellulaseSS6/control_ = 87.203, *p*_cellulaseSS6/control_ = 0.001, F_cellulaseNS6/control_ = 79.853, *p*_cellulaseNS6/control_ = 0.001, F_cellulaseSS6/AA_ = 69.334, *p*_cellulaseSS6/AA_ = 0.001, F_cellulaseNS6/AA_ = 65.471, *p*_cellulaseNS6/AA_ = 0.001, F_cellulaseSS6/CTS_ = 85.348, *p*_cellulaseSS6/CTS_ = 0.001, F_cellulaseNS6/CTS_ = 77.136, *p*_cellulaseNS6/CTS_ = 0.001, F_cellulaseSS6/CTS+AA_ = 70.773, *p*_cellulaseSS6/CTS+AA_ = 0.001, F_cellulaseNS6/CTS+AA_ = 65.305, *p*_cellulaseNS6/CTS+AA_ = 0.001; F_glucoamylaseSS6/control_ = 16.454, *p*_glucoamylaseSS6/control_ = 0.015, F_glucoamylaseNS6/control_ = 16.782, *p*_glucoamylaseNS6/control_ = 0.015, F_glucoamylaseSS6/AA_ = 16.202, *p*_glucoamylaseSS6/AA_ = 0.016, F_glucoamylaseNS6/AA_ = 16.508, *p*_glucoamylaseNS6/AA_ = 0.015, F_glucoamylaseSS6/CTS_ = 12.522, *p*_glucoamylaseSS6/CTS_ = 0.024, F_glucoamylaseNS6/CTS_ = 13.021, *p*_glucoamylaseNS6/CTS_ = 0.023, F_glucoamylaseSS6/CTS+AA_ = 9.776, *p*_glucoamylaseSS6/CTS+AA_ = 0.035, F_glucoamylaseNS6/CTS+AA_ = 10.389, *p*_glucoamylaseNS6/CTS+AA_ = 0.032; F_pectinaseSS6/control_ = 65.523, *p*_pectinaseSS6/control_ = 0.001, F_pectinaseNS6/control_ = 82.157, *p*_pectinaseNS6/control_ = 0.001, F_pectinaseSS6/AA_ = 70.167, *p*_pectinaseSS6/AA_ = 0.001, F_pectinaseNS6/AA_ = 88.994, *p*_pectinaseNS6/AA_ = 0.001, F_pectinaseSS6/CTS_ = 43.306, *p*_pectinaseSS6/CTS_ = 0.003, F_pectinaseNS6/CTS_ = 54.594, *p*_pectinaseNS6/CTS_ = 0.002, F_pectinaseSS6/CTS+AA_ = 40.891, *p*_pectinaseSS6/CTS+AA_ = 0.003, F_pectinaseNS6/CTS+AA_ = 51.624, *p*_pectinaseNS6/CTS+AA_ = 0.002) and higher than those of SS2, SS4, SS10, NS2, NS4 and NS10 to some extent, but there was no marked differences in the neutral protease, acid protease, α-amylase, cellulase, glucoamylase and pectinase activities between SS6 and NS6 (F_neutralproteaseSS6/NS6_ = 0.803, *p*_neutralproteaseSS6/NS6_ = 0.421; F_acidproteaseSS6/NS6_ = 1.799, *p*_acidproteaseSS6/NS6_ = 0.251; F_α−*amylaseSS*6/*NS*6_ = 0.358, *p*_α−*amylaseSS*6/*NS*6_ = 0.582; F_cellulaseSS6/NS6_ = 0.287, *p*_cellulaseSS6/NS6_ = 0.626; F_glucoamylaseSS6/NS6_ = 0.057, *p*_glucoamylaseSS6/NS6_ = 0.823; F_pectinaseSS6/NS6_ = 0.386, *p*_pectinaseSS6/NS6_ = 0.568). The above analyses indicated the optimal concentration of nano-selenium used in soybean soaking was 6 mg/L. An appropriate concentration of selenium could effectively maintain the integrity of microbial cells and improve the activities of selenium dependent enzymes in microorganisms, so as to promote the growth of microorganisms and secrete more enzymes. However, once the concentration of selenium is excessively high (especially more than 10 mg/L), selenium exhibits toxicity, which leads to oxidative stress of microorganisms. A large number of reactive oxygen free radicals would destroy the metabolic enzyme system of microorganisms, resulting in the loss of protein activity, thus inhibiting the growth of microorganisms (33).

**Table 5:** Effects of selenium on enzyme activities in kojis.

	**Acid protease activities (U/g)**	**Neutral protease activities (U/g)**	**α-Amylase activities (U/g)**	**Glucamylase activities (U/g)**	**Cellulase activities (U/g)**	**Pectinase activities (U/g)**
Control	185.34 ± 5.45^e^	1,156.52 ± 33.07^b^	165.14 ± 4.24^d^	1,812.57 ± 59.25^e^	240.17 ± 8.16^d^	204.43 ± 6.70^f^
AA	188.08 ± 6.22^e^	1,161.21 ± 33.16^b^	167.42 ± 4.86^c, d^	1,819.23 ± 55.84^d, e^	243.56 ± 9.47^d^	205.27 ± 5.87^f^
CTS	190.42 ± 5.83^d, e^	1,163.68 ± 31.28^b^	168.54 ± 5.06^c, d^	1,836.93 ± 61.68^d, e^	244.40 ± 7.08^d^	209.66 ± 8.14^e, f^
AA+CTS	191.99 ± 5.16^d, e^	1,168.53 ± 26.73^b^	168.83 ± 5.39^c, d^	1,842.16 ± 75.31^c, d, e^	248.58 ± 7.66^d^	210.39 ±8.32^e, f^
SS2	196.76 ± 4.52^d, e^	1,175.78 ± 35.77^a, b^	174.90 ± 4.34^c, d^	1,896.34 ± 67.99^c, d, e^	267.29 ± 10.58^c^	217.62 ± 7.78^e, f^
SS4	209.14 ± 8.63^b, c^	1,202.62 ± 36.99^a, b^	189.28 ± 6.20^b^	1,948.40 ± 88.15^a, b, c, d^	282.59 ± 9.74^b, c^	231.81 ± 8.19^c, d^
SS6	235.06 ± 9.75^a^	1,240.26 ± 36.01^a^	216.46 ± 8.41^a^	2,028.69 ± 70.75^a, b^	309.84 ± 10.02^a^	252.52 ± 7.81^a, b^
SS10	186.84 ± 4.58^e^	1,159.96 ± 37.91^b^	166.07 ± 5.31^d^	1,813.75 ± 70.20^e^	242.27 ± 8.61^d^	205.80 ± 8.72^f^
NS2	200.37 ± 6.02^c, d^	1,181.35 ± 45.50^a, b^	177.58 ± 4.37^c^	1,914.62 ± 56.71^b, c, d, e^	273.73 ± 7.82^c^	223.64 ± 6.96^d, e^
NS4	218.22 ± 5.71^b^	1,215.67 ± 48.58^a, b^	195.00 ± 5.71^b^	1,968.94 ± 65.65^a, b, c^	290.90 ± 9.06^b^	240.93 ± 7.38^b, c^
NS6	244.60 ± 7.53^a^	1,269.38 ± 43.25^a^	220.61 ± 8.59^a^	2,043.15 ± 77.42^a^	314.57 ± 11.89^a^	256.36 ± 7.32^a^
NS10	190.98 ± 4.87^d, e^	1,163.39 ± 38.75^a, b^	167.34 ± 4.29^c, d^	1,823.47 ± 63.01^d, e^	248.72 ± 8.08^d^	211.70 ± 6.72^e, f^

^a−f^* Different letters in the same column indicate the significant differences (p < 0.05)*.

Proteases secreted by *Aspergillus oryzae*, especially acid proteases, could degrade proteins into peptides, oligopeptides and amino acids (36). Basically, the FAAs content in soy sauce was positively related to the activity of proteases in kojis. *A. oryzae* can also secrete cellulase and pectinase to degrade the soybean cell wall components, so that the total phenolics and total flavonoids attached to the soybean cell wall in the form of esterification or insoluble binding were released from the cell wall (16). Thus, the increased cellulase and pectinase activities could be of help to the release of total phenolics and total flavonoids from the soybean cell wall.

Combined with the above analyses, it could be concluded that appropriate content of selenium used during soybean soaking could promote the growth of *A. oryzae* and improve the activities of neutral protease, acid protease, α-amylase, cellulase, glucoamylase and pectinase, which were beneficial to increasing the contents of small peptides, free amino acids, Maillard reaction products, total phenolics and total flavonoids in soy sauces, so as to improve the antioxidant activities of soy sauce.

### Cluster analysis

**Figure 3:**
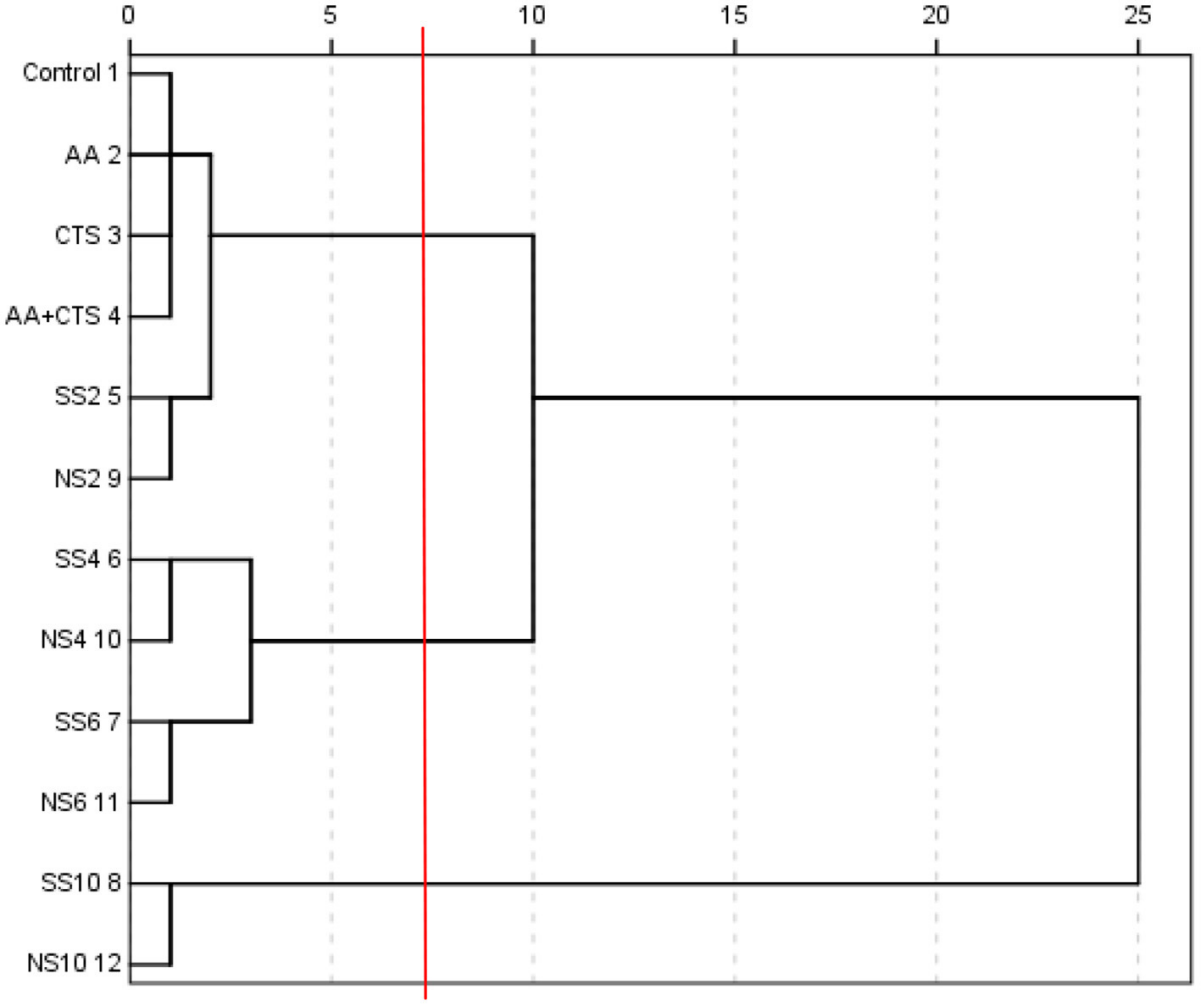
Result of cluster analysis of soy sauces.

Four indices of antioxidant activities (DRSA, ARSA, RP and MICA) and 12 active ingredients (TP, TF, FN, TTA, RS, TS, MRP, NSS, TSe, ISe, OSe and FAAs) of soy sauces were systematically clustered according to the square euclidean distance, the results were shown in Figure 3.

When the distance was more than 5 and less than 10 (i.e., the red line in Figure 3), soy sauces could be well divided into three categories, among which NS6, SS6, NS4 and SS4 were the first category (C1), NS10 and SS10 were the second category (C2), and NS2, SS2, AA+CTS, CTS, AA and the control were the third category (C3). It can be concluded that group C1 had the strongest antioxidant activity, followed by group C2 and C3 according to the above analyses.

The results suggested that low concentration of nano-selenium and sodium selenite, ascorbic acid and chitosan contained in nano-selenium had no significant effects on the release and formation of various active ingredients and the antioxidant activities of soy sauces. Therefore, NS2, SS2, AA+CTS, CTS, AA and the control were classified into the same category (C3). The appropriate concentration of selenium could increase the contents of selenium and various active ingredients in soy sauces, so as to improve the antioxidant activities of soy sauces. Therefore, NS6, SS6, NS4 and SS4 were classified into the same category (C1). Interestingly, nano-selenium and sodium selenite with the same concentration in C1, C2 and C3 were all divided into the same smallest categories, indicating the insignificant effects of nano-selenium and sodium selenite with the same concentration on the antioxidant activities of soy sauces.

### Principal component analysis

Principal component analysis was conducted on the original data of 16 variables including antioxidant activities (DRSA, ARSA, RP and MICA) and active ingredients (TP, TF, FN, TTA, RS, TS, MRP, NSS, TSe, ISe, OSe and FAAs). In order to ensure the integrity and reliability of information, the cumulative variance contribution rate should be over 80%, and the results were shown in Tables 6–8.

**Table 6:** Initial eigenvalue and cumulative variance contribution rate of principal components.

**Principal component**	**Characteristic value**	**Variance contribution rate %**	**Cumulative variance contribution rate %**
1	12.712	79.452	79.452
2	2.940	18.372	97.825
3	0.153	0.955	98.780
4	0.094	0.587	99.367
5	0.042	0.260	99.627
6	0.030	0.188	99.815
7	0.016	0.101	99.916
8	0.006	0.037	99.953
9	0.006	0.035	99.988
10	0.002	0.009	99.997
11	0.000	0.003	100.000
12	1.561E-16	9.753E-16	100.000
13	1.096E-16	6.849E-16	100.000
14	−8.718E-18	−5.449E-17	100.000
15	−7.695E-17	−4.809E-16	100.000
16	−3.770E-16	−2.356E-15	100.000

**Table 7:** Source of variation for the principal component (PC).

**Item**	**First principal component (PC1)**	**Second principal component (PC2)**
Total phenolics	0.996	−0.003
Total flavonoids	0.969	0.019
Formaldehyde nitrogen	0.991	0.020
Total titratable acid	0.991	−0.094
Reducing sugar	0.959	−0.192
Total sugar	0.981	−0.065
Maillard reaction products	0.967	−0.070
Non-salt soluble solids	0.992	−0.030
Total selenium	0.254	0.966
Inorganic selenium	0.112	0.993
Organic selenium	0.268	0.962
Free amino acids	0.987	−0.146
DPPH radical scavenging activity	0.996	−0.041
ABTS radical scavenging activity	0.977	−0.097
Reducing power	0.982	0.080
Metal ion chelating activity	0.992	−0.013

**Table 8:** Main component score and comprehensive score of soy sauces.

**Number**	**Sample name**	**PC1 score**	**PC2 score**	**Comprehensive score**	**Comprehensive score ranking**
1	Control	−3.24971	−1.25630	−2.87528	12
2	AA	−3.14403	−1.27655	−2.79325	11
3	CTS	−2.53603	−1.34253	−2.31185	10
4	AA+CTS	−2.25557	−1.37982	−2.09108	9
5	SS2	−1.05910	−0.70342	−0.99229	6
6	SS4	1.91351	−0.08098	1.53887	4
7	SS6	5.50058	0.62814	4.58536	2
8	SS10	−3.07821	3.82273	−1.78197	8
9	NS2	−0.24238	−0.90597	−0.36702	5
10	NS4	3.12278	−0.54891	2.43311	3
11	NS6	7.03630	0.07910	5.72949	1
12	NS10	−2.00810	2.96453	−1.07406	7

According to Table 6, the cumulative variance contribution rate was 97.83%, and the eigenvalues of the first two principal components were higher than 1, thus the first two principal components were extracted. Among them, the eigenvalue of the first principal component (PC1) was 12.71, which was the most important factor, explaining 79.45% of the variation. As shown in Table 7, FN, TTA, RS, TS, MRP, NSS, FAAs, TP, TF, DRSA, ARSA, RP and MICA had a high load on PC1, indicating that PC1 primarily represented the information of these indicators. The eigenvalue of the second principal component (PC2) was 2.94, PC2 explained 18.37% of the variation. As shown in Table 7, TSe, ISe and OSe had a high load on PC2, indicating that PC2 primarily represented the information of these indicators. Since the extraction of the first two principal components could primarily represent the information of all indicators, the two new variables (PC1 and PC2) were used to replace the original 16 variables. PC1 score, PC2 score and comprehensive score were calculated. As exhibited in Table 8, the results demonstrated that the comprehensive score of NS6 ranked 1, followed by SS6, NS4, SS4, NS2, SS2, NS10, SS10, AA+CTS, CTS, AA and the control.

The above principal component analysis confirmed again that the appropriate concentration of selenium not only increased the contents of total selenium and organic selenium in soy sauce, but also enhanced the soy sauce's antioxidant activities.

## Conclusion

In light of the above analyses, compared with the control, the appropriate concentration of selenium (6 mg/L nano-selenium) could increase the contents of total selenium and organic selenium in soy sauce significantly, promote the growth and metabolism of *A. oryzae*, and make the latter secrete more proteases, amylases and other enzymes. The enzymes were beneficial to promoting the release and formation of antioxidant compounds including phenolics, flavonoids, free amino acids and Maillard reaction products during moromi fermentation, and the latter enhanced the antioxidant activities of soy sauce. In addition, in view of nano-selenium had lower toxicity and higher biological activities than sodium selenite, nano-selenium may have a broader application prospect in selenium-enriched soy sauce industry in the future. In conclusion, the work developed a widely accessible selenium supplement and a novel method to enhance organic selenium content and antioxidant activities of soy sauce and clarified the related mechanisms. Further work on the effects of nano-selenium on aroma and taste of soy sauce and the purification and identification of selenium-containing peptides with strong antioxidant activities in soy sauce is in progress.

## Data availability statement

The raw data supporting the conclusions of this article will be made available by the authors, without undue reservation.

## Author contributions

JC and TF involved in investigation, data curation, formal analysis, and writing original draft. BW contributed to conceptualization, visualization, resources, and writing review and editing. RH performed methodology, supervision, validation, and writing review and editing. YX and PG involved in investigation and formal analysis. Z-HZ and LZ contributed to conceptualization, methodology, resources, and visualization. JF and ZL involved in conceptualization, formal analysis, validation, and resources. XG contributed to conceptualization, project administration, methodology, resources, supervision, and writing review and editing. All authors contributed to the article and approved the submitted version.

## Funding

The authors declare that this study only received funding from the National Natural Science Foundation of China (32101893, 31801537), the Special Fund for Science and Technology Development of Zhongshan, China (2018A1007), the Science and Technology Innovation Strategy Special Project of Yangxi, China (SDZX2021030). The funders were not involved in the study design, collection, analysis, interpretation of data, the writing of this article or the decision to submit it for publication.

## Conflict of interest

Authors JF and ZL were employed by Guangdong Chubang Food Co., Yangjiang, China. The remaining authors declare that the research was conducted in the absence of any commercial or financial relationships that could be construed as a potential conflict of interest.

## Publisher's note

All claims expressed in this article are solely those of the authors and do not necessarily represent those of their affiliated organizations, or those of the publisher, the editors and the reviewers. Any product that may be evaluated in this article, or claim that may be made by its manufacturer, is not guaranteed or endorsed by the publisher.
